# Post Self-Crosslinking of Phthalonitrile-Terminated Polyarylene Ether Nitrile Crystals

**DOI:** 10.3390/polym10060640

**Published:** 2018-06-08

**Authors:** Lifen Tong, Renbo Wei, Yong You, Xiaobo Liu

**Affiliations:** Research Branch of Advanced Functional Materials, School of Materials and Energy, University of Electronic Science and Technology of China, Chengdu 610054, China; weirb10@uestc.edu.cn (R.W.); yourkeaib@163.com(Y.Y.)

**Keywords:** kinetics, crosslinking, crystallization, activation energy, differential scanning calorimetry, polyaryl ether nitrile

## Abstract

A novel phthalonitrile-terminated polyaryl ether nitrile (PEN-Ph) was synthesized and characterized. The crystallization behavior coexisting with the crosslinking behavior in the PEN-Ph system was confirmed by rheological measurements. DSC was applied to study the crystallization kinetics and crosslinking reaction kinetics. Through the Avrami equation modified by Jeziorny, the nonisothermal crystallization kinetics were analyzed, and the Avrami exponent of about 2.2 was obtained. The analysis results of more intuitive polaring optical microscopy (POM) and SEM indicated that the shape of the crystals is similar to spherical. Moreover, the activation energy of the crystallization behavior and crosslinking behavior were obtained by the Kissinger method, and the values were about 152.7 kJ·mol^−1^ and 174.8 kJ·mol^−1^, respectively. This suggests that the activation energy of the crystallization behavior is lower than that of the crosslinking behavior, indicating that the crystallization behavior is more likely to occur than the crosslinking behavior and the crystals of PEN-Ph can be self-crosslinked to form single-polymer composites.

## 1. Introduction

Single-polymer composites, also described as homocomposites, one-polymer composites, self-reinforced, all (the same) polymer composites, or homogeneous composites, in which both reinforcement and matrix are from the same polymer, have attracted considerable interest in the academic field due to their excellent performance characteristics such as light weight, ideal recyclability, and superior mechanical properties [1,2,3,4,5]. In a previous report, most studied systems are based on the use of polyolefin and polyester materials [6]. Various techniques were applied to design and produce single-polymer composites such as overheating, solution, partial dissolving, cool drawing, physical treatment, and chemical modification. Among these methods, film stacking is most frequently used, in which the chosen matrix film generally has a lower melting point than the fibers, so that only the interleaved film melts.

As a well-known type of high-performance thermoplastic resin, polyarylene ether nitriles (PENs) have triggered considerable activity in material synthesis and characterization as well as theoretical studies in the thermal, mechanical, and electric fields. Because of the aryl ether bond in the polymer main chain, PEN possesses outstanding properties such as excellent thermal and thermo-oxidative stability, good mechanical properties, superior chemical inertia, and radiation resistance [7,8,9,10,11,12]. In our previous paper, a novel phthalonitrile-terminated polyarylene ether nitrile (PEN-Ph) was synthesized through a two-step, one-pot method [13,14]. Compared with the conventional PEN, PEN-Ph has crosslinkable phthalonitrile at the end of the main chain, which endows it with the crosslinkable property.

Because of the certain regular structure of the molecular chain and the phthalonitrile groups at the end of the molecular chain, PEN-Ph possesses crystallization properties combined with crosslinking properties [14,15]. In this work, the crystallization behavior and crosslinking behavior were studied. From the Jeziorny method, the Avrami exponent and crystallization rate constants could be obtained. Besides this, the crystallization activation energy and crosslinking activation energy were obtained through the Kissinger method, and, thus, the question about which of the crystallization behavior and crosslinking behavior occurs more easily could be answered by comparing the activation energies of the crystallization behavior and crosslinking reaction. Most importantly, a novel single-polymer composite based on PEN-Ph was prepared through a simple hot-pressing method in which the post solid-state crosslinking of the PEN-Ph crystals occurs. Firstly, the PEN-Ph crystals formed at an appropriate temperature. Then, the phthalonitrile groups on the crystal surface reacted with the amorphous PEN-Ph to form thermally stable triazine rings and phthalocyanine rings, so that the PEN-Ph crystals could be solidified through a crosslinking reaction. Therefore, in this PEN-Ph single-polymer composite, the crystals and physical crosslinking points acted as reinforcement to improve the mechanical and thermal properties of the material.

## 2. Materials and Methods 

### 2.1. Materials

**Figure 1:**
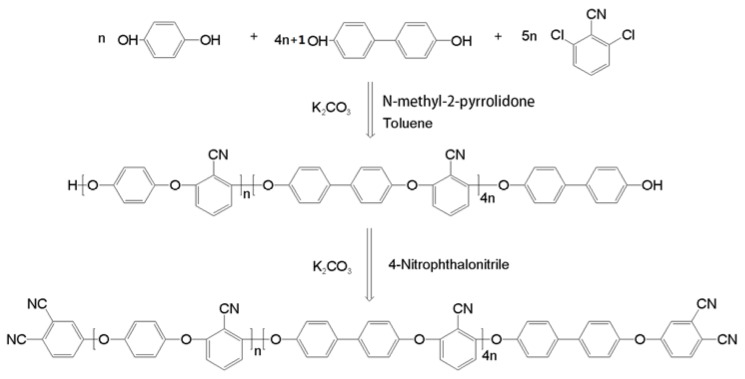
Synthetic route of phthalonitrile-terminated polyaryl ether nitrile (PEN-Ph).

*N*-methyl-2-pyrrolidone (NMP) and toluene were purchased from Tianjin BODI Chemicals, Tianjin, China. Hydroquinone (HQ), biphenol (BP), 2,6-dichlorobenzonitrile (DCBN), and K_2_CO_3_ were supplied by Alpha Chemicals Co. Ltd., Dezhou, China. The phthalonitrile end-capped polyarylene ether nitrile was synthesized through a two-step, one-pot method. Firstly, the hydroxyl end-capped polyarylene ether nitrile (PEN-OH) was synthesized from HQ, BP, and DCBN by nucleophilic polycondensation reaction in the presence of K_2_CO_3_ as catalyst, NMP as polar solvent, and toluene as dehydrating agent. Secondly, the PEN-OH was end-capped by the 4-nitrophthalonitrile to obtain the PEN-Ph [16]. Then, the obtained PEN-Ph mixture was poured into a beaker filled with butanone to remove small molecules and then filtrated to retrieve the PEN-Ph powder. Finally, the PEN-Ph product was purified with dilute HCl solution, deionized water, and alcohol until the solvent and small molecules were washed away completely, and then dried at 120 °C overnight. The detail of the synthetic route of the PEN-Ph is shown in Figure 1.

### 2.2. Measurements and Characterization Techniques

The molecular weight of the PEN-Ph was measured using gel permeation chromatography (GPC) which was conducted with a PL-GPC220 system using polystyrene as the standard and THF as the eluent. The number-averaged molecular weight (Mn) of the PEN-Ph is 9.4 × 10^3^ g/mol, and the Polymer dispersity index is 2.401.

Rheological analysis was performed using a TA Instruments Rheometer AR-G2. Differential scanning calorimetry (DSC) was performed on a TA Instruments DSC-Q100 under a nitrogen flow rate of 50 mL/min from 80 °C to 350 °C. X-ray powder diffraction (XRD, Rigaku RINT2400 with Cu Kα radiation) was employed to confirm the formation of the PEN-Ph crystals. The micromorphologies of the PEN-Ph crystals were characterized by scanning electron microscopy (SEM) (JSM 6290LV). For optical microscopy observation, an MP41 optical microscope was used in this study. All optical micrographs presented in this paper were taken under a crossed polarizer.

## 3. Results and Discussion

### 3.1. Rheological Properties

**Figure 2:**
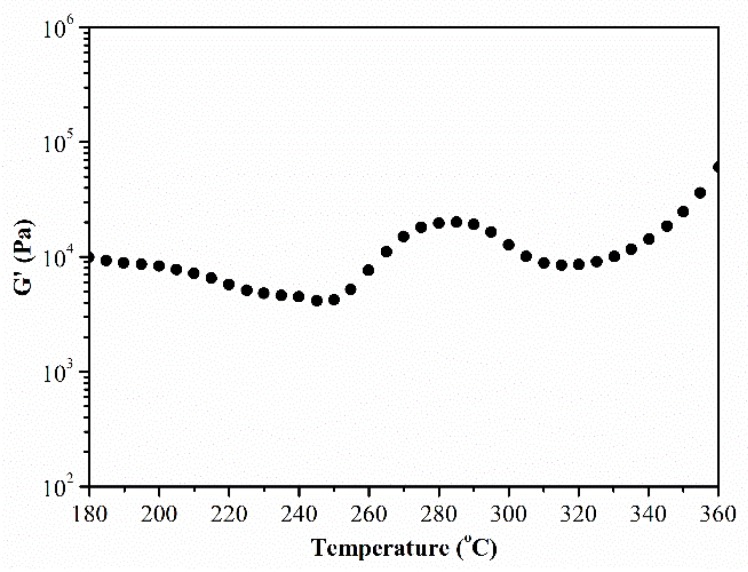
Rheological properties as a function of temperature.

The physical and chemical changes of the polymers can be reflected efficiently through rheological testing. In order to investigate the properties of the PEN-Ph, rheological testing was employed. Figure 2 shows the rheological curve of temperature scanning in the range of 180 °C to 360 °C. A slight decrease of the storage modulus (G’) at the temperature of about 200 °C is mainly caused by chain segment motion, and this temperature is the glass transition temperature, which is determined from the volume changes during heating of the PEN-Ph systems [17,18]. When the temperature rises to about 250 °C, G’ starts to trend upwards. The first increase of G’ is mainly due to the formation of the PEN-Ph crystals. Then, the G’ trend declines with temperature increase to about 290 °C, which is caused by the PEN-Ph crystals melting and the sliding between the polymer chains when the temperature increases while the crosslinking process is not activated. As the temperature further increases, the G’ increases once more. The second increase of G’ is mainly caused by the crosslinking reaction among the PEN-Ph to form a network structure. These results proved that the PEN-Ph possesses a crosslinking reaction combined with crystallization behavior.

### 3.2. XRD Analysis

**Figure 3:**
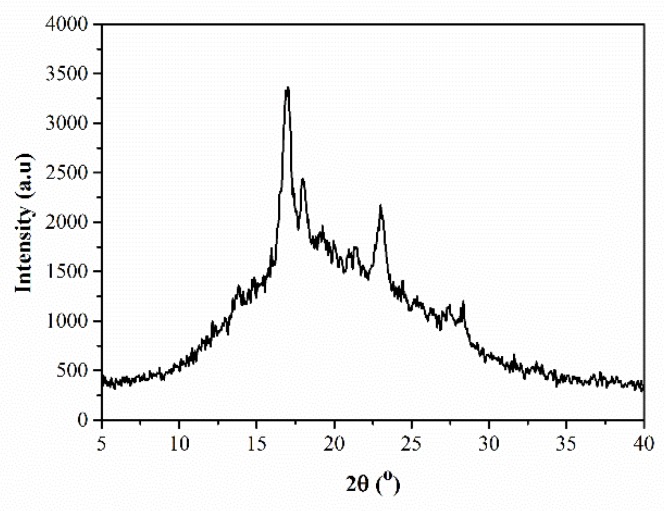
XRD pattern of the PEN-Ph system.

From Figure 1, it can be seen that the PEN-Ph main chain has a certain degree of regularity, leading to the crystallization behavior of PEN-Ph. The crystallization behavior of PEN-Ph could be further investigated using XRD, and the result is shown in Figure 3. The measured PEN-Ph sample was prepared through the hot-pressing method at 300 °C under 10 MPa for 4 h. From the XRD pattern, it can be clearly observed that there is a steamed-bun-like peak in the range of 10–30° which is attributed to the amorphous background resin. Furthermore, there are three obvious diffraction peaks in the curve at 17.0°, 18.2°, and 23.3°. The presence of these three obvious diffraction peaks proves the existence of PEN-Ph crystals. Moreover, the crystallinity is calculated to be 18.2% through peak-differentiating and imitating. These results provide evidence of the crystallization in the PEN-Ph system.

### 3.3. Nonisothermal Crystallization Kinetics

To investigate the crystallization behavior of PEN-Ph, the nonisothermal crystallization kinetics of PEN-Ph were studied [19,20,21,22]. Based on the Avrami equation, Jeziorny postulated that the increasing or decreasing of temperature at an equal rate is an isothermal process, and he applied the Avrami equation to the analysis of DSC curves to obtain the Avrami exponent and parameter K_t_ [23]. Therefore, the Avrami equation is modified as below.

(1) $$1-X_{t}=\exp\left(-K_{t}t^{n}\right)$$

Taking double logarithms, Equation (1) is transformed into

(2) $$\ln[-\ln\left(1-X_{t}\right)]={\mathrm{lnK}}_{t}+\mathrm{nlnt}$$

where t is the crystallization time; the exponent n is a mechanism constant, the value of which depends on the nucleation mechanism and growth dimensions; K_t_ is the growth rate constant, which depends on nucleation and crystal growth; and X_t_ is the relative degree of crystallinity, which can be defined as follows [24]:

(3) $$X_{t}=\frac{\int_{T_{0}}^{T}\frac{\partial H_{c}}{\partial T}\mathrm{dt}}{\int_{T_{0}}^{T_{\infty }}\frac{\partial H_{c}}{\partial T}\mathrm{dt}}$$

where T_0_ and T_∞_ represent the onset and end crystallization temperatures, respectively. Therefore, the Avrami exponent n can be obtained from the slope of the plot of ln[−ln(1 − X_t_)] versus lnt, and K_t_ can be obtained from the intercept of the plot of ln[−ln(1 − X_t_)] versus lnt. Then, the crystallization rate constant K_t_ is modified by the below equation, where β is the heating rate or cooling rate.

(4) $$\ln K_{c}=\frac{\ln K_{t}}{\beta }$$

**Figure 4:**
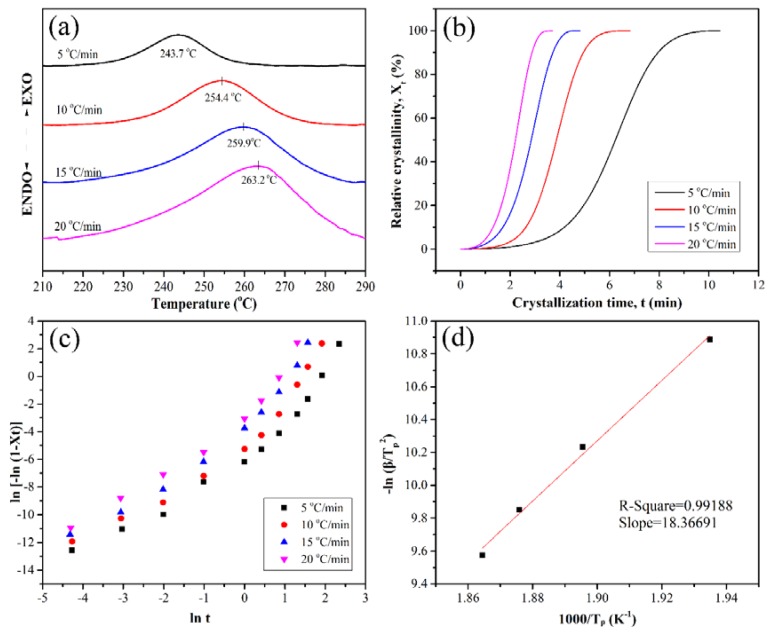
Crystallization behavior of the PEN-Ph.

Figure 4a depicts the DSC curves of the PEN-Ph with different heating rates. It can be seen that all the curves have a cold crystallization peak in the range of 210 °C to 290 °C. With the heating rate increasing, the crystallization peak temperature (T_p_) increases from 243.7 °C to 263.2 °C. The shift of the cold crystallization peak is mainly due to the fact that the polymeric molecular chain is a peristalsis process of chain segments which is driven by diffusion during the crystallization process [25].

In the studies of the nonisothermal crystallization kinetics, the relative degree of crystallinity (X_t_) is an important parameter. Figure 4b shows the relative degree of crystallinity as a function of the crystallization time, which can be obtained by partial integration of the crystallization exotherm’s peak. It can be clearly observed that all the curves of X_t_ versus t with various heating rates have the same characteristic sigmoid shape. The sigmoid shape can be divided into three parts, which reflect the three parts of the crystallization process, respectively. In the first part, X_t_ exhibits a nonlinear relationship with time, which is generally considered to be the nucleation step; in the second part, X_t_ exhibits a linear relationship with time, which is considered to be primary crystallization; the third part is another nonlinear part, which is caused by the spherulitic impregnation in the late stage of the crystal growth, and it is considered to be secondary crystallization [22]. Furthermore, from the X_t_ versus t curves with various heating rates, it is clear that the higher the heating rate is, the shorter time the crystallization needs.

Figure 4c displays the plots of ln[−ln(1 − X_t_)] versus lnt at different heating rates. Through simulating the plots, the slopes and intercept were obtained; detailed data are listed in Table 1. The Avrami exponents n are 2.12, 2.22, 2.28, and 2.23 at the heating rates of 5, 10, 15, and 20 °C·min^−1^, respectively. The values of the Avrami exponent indicate that the crystals’ shape was between disk and sphere, which can be confirmed by the polarizing microscope photographs. As the heating rate increased, the crystallization rate constant K_c_ increased from 0.37 to 0.90.

Furthermore, the Kissinger method has also been previously employed to calculate the activation energy of the crystallization process [26,27]. From Equation (5), the activation energy E_α_ can be obtained from the slope of the plot of $-\ln\left(\frac{\beta }{T_{p}^{2}}\right)$ versus 1000/T_p_, which is displayed in Figure 4d.

(5) $$\ln\left(\frac{\beta }{T_{p}^{2}}\right)=\mathrm{Const}-\left(\frac{E_{\alpha }}{{\mathrm{RT}}_{p}}\right)$$

Through linear fitting, the slope is obtained at 18.367. Then, the activation energy of the crystallization process is obtained to be 152.7 kJ·mol^−1^ through calculation.

### 3.4. Crosslinking Reaction Kinetics

Because of the crosslinkable property of the phthalonitrile at the end of the PEN-Ph chain, a crosslinking reaction can occur among the PEN-Ph molecules. In order to investigate the difficulty of the crosslinking reaction, the reaction kinetics of the PEN-Ph matrix were studied. Doyle et al. obtained the reaction kinetics equation as follows through data analysis [28].

(6) $$\ln\beta =\mathrm{Const}-1.052\left(\frac{E_{\alpha }}{{\mathrm{RT}}_{\alpha }}\right)$$

However, the activation energy calculated by this method has some variances. Kissinger came up with a new parameter for the reaction kinetics equation [26]. Thus, the reaction kinetics equation was obtained as follows:

(7) $$\ln\left(\frac{\beta }{T_{p}^{2}}\right)=\mathrm{Const}-\left(\frac{E_{\alpha }}{{\mathrm{RT}}_{p}}\right).$$

**Figure 5:**
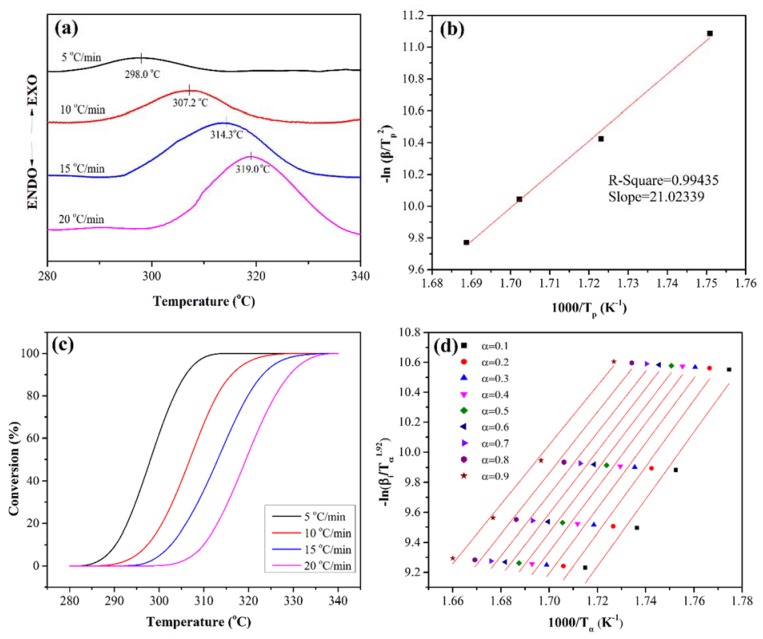
Crosslinking reaction behavior of the PEN-Ph.

Figure 5a shows the DSC curves of the PEN-Ph under different scan heating rates. It can be clearly observed that there is an exothermic peak in the range of 280 °C to 340 °C in all the DSC curves. This exothermic peak is caused by the crosslinking reaction of the phthalonitrile at the end of the PEN-Ph chain. With the heating rates increases, the exothermic peak shifts to a higher temperature and the enthalpy of the exothermic peak increases. This is mainly due to the fact that the polymeric molecular chain is a peristalsis process of chain segments which is driven by diffusion during the crosslinking process.

Figure 5b shows the values of $-\ln\left(\frac{\beta }{T_{p}^{2}}\right)$ as a function of 1000/T_p_. Through linear fitting, the slope of the plot of $-\ln\left(\frac{\beta }{T_{p}^{2}}\right)$ versus 1000/T_p_ was obtained at 21.023. Then, the activation energy of the crystallization process was obtained to be 174.8 kJ·mol^−1^ through calculation.

Then, the reaction kinetics equation was further modified by Starink et al. through the Conversion method as follows [29]:

(8) $$\ln\left(\frac{\beta }{T_{\alpha }^{1.92}}\right)=\mathrm{Const}-1.008\left(\frac{E_{\alpha }}{{\mathrm{RT}}_{\alpha }}\right).$$

The accuracy of the activation energy calculated by this equation is improved.

The relationship between the conversion rate and enthalpy can be expressed in the below equation:

(9) $$\alpha =\frac{\int_{T_{0}}^{T}\frac{\partial H}{\partial T}\mathrm{dT}}{\int_{T_{0}}^{T_{\infty }}\frac{\partial H}{\partial T}\mathrm{dT}}$$

where T_0_ and T_∞_ represent the onset and end crosslinking reaction temperatures, respectively. ∂H/∂T is the heat capacity. Figure 5c shows the curves of the conversion as a function of the temperature. It can be seen that all the curves have the same characteristic sigmoid shape, indicating that the curing reaction is an autocatalytic reaction.

The temperature at various conversions (T_α_) can be obtained through the above equation. Then, the relationship between $\ln\left(\frac{\beta }{T_{\alpha }^{1.92}}\right)$ and 1000/T_α_ can be obtained, which is shown in Figure 5d. The activation energy at various conversions can be calculated from the slopes of the fitting curves, and the detailed data are listed in Table 2. The average value of the E_α_ was 175.23 kJ·mol^−1^, which is close to the E_α_ calculated by the Kissinger method.

### 3.5. Morphology Properties

**Figure 6:**
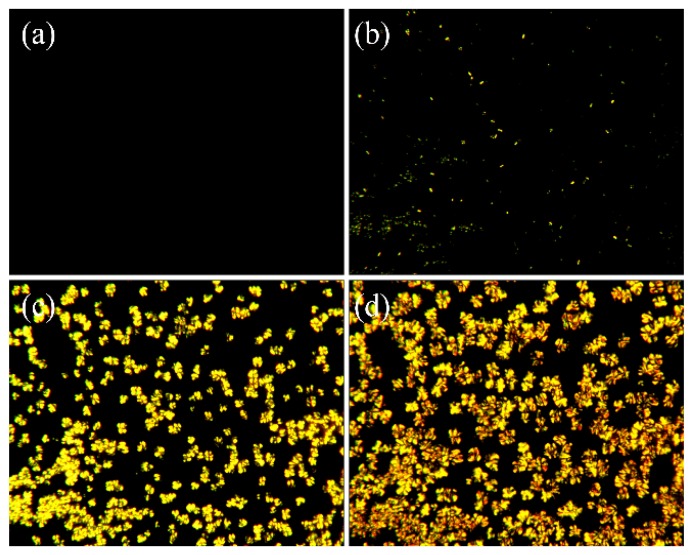
Polaring optical microscopy (POM) of the process of growth of the PEN-Ph crystals at 280 °C. (**a**) 0 min; (**b**) 10 min; (**c**) 30 min; (**d**) 60 min.

**Figure 7:**
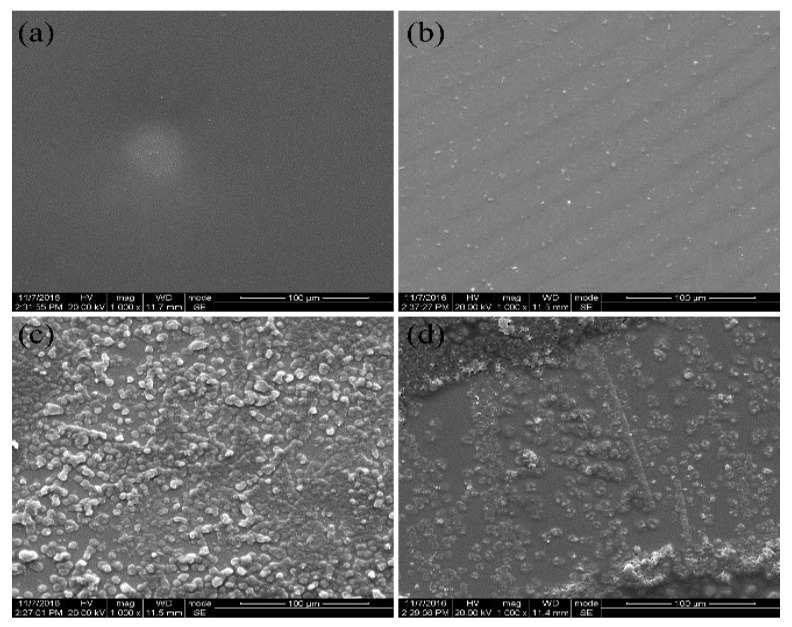
SEM of the process of growth of the PEN-Ph crystals at 280 °C. (**a**) 0 min; (**b**) 10 min; (**c**) 30 min; (**d**) 60 min.

From the analysis of the crystallization kinetics, the Avrami exponent that is related to the nucleation mechanism and the mode of crystal growth was obtained. polaring optical microscopy (POM) was provided to characterize the morphology of the PEN-Ph crystals. Figure 6 shows the POM of the growing process of the PEN-Ph crystals. It can be seen from Figure 6a that there is only darkness in the screen, indicating that there are not any crystals in the sample. Then, some small crystals appear in the sample on the heating stage for 10 min (Figure 6b). With the heating time increasing, the PEN-Ph crystals grew larger, appearing similar to a Maltese cross in the PEN-Ph crystals, indicating that the shape of the PEN-Ph crystals is similar to a sphere. In order to further confirm the morphology of the PEN-Ph crystals, SEM was employed, and the results are shown in Figure 7. The phenomenon of PEN-Ph crystal growth observed from SEM is the same trend as that observed from POM. From Figure 7c, it can be clearly observed that the PEN-Ph crystals’ shape is similar to spherical, which well agrees with the analysis results of the POM and crystallization kinetics.

### 3.6. Thermal and Mechanical Properties

In the crosslinkable PEN-Ph system, the thermal and mechanical properties can be enhanced after heat treatment because of the crosslinking network formation. Therefore, samples of amorphous PEN-Ph and PEN-Ph single-polymer composites with similar crosslinking degree were prepared though the hot-pressing method and their properties were compared; this is shown in Table 3. It can be seen that the T_g_ of the single-polymer composites is 204.6 °C, an increase of about 6.0 °C over that of the amorphous PEN-Ph sheet, and the tensile strength of the single-polymer composites is 111.0 MPa, an increase of about 10 MPa over that of the amorphous PEN-Ph sheet. Hence, we draws the conclusion that PEN-Ph single-polymer composites possess better thermal and mechanical properties than amorphous PEN-Ph.

## 4. Conclusions

In summary, crystallization behavior coexists with crosslinking reaction in the PEN-Ph system because of the regular main chain and the crosslinkable phthalonitrile at the end of the PEN-Ph main chain. Therefore, the crystallization kinetics and crosslinking reaction kinetics were studied. Through the Avrami equation modified by Jeziorny, the nonisothermal crystallization kinetics were analyzed, and the Avrami exponent were determined to be about 2.2, demonstrating that the shape of the crystals is between disk and sphere. From POM observation, we draw the conclusion that the PEN-Ph crystals grew bigger as the heating time increased and appear similar to a Maltese cross, indicating that the shape of PEN-Ph crystals is similar to a sphere. The SEM observation results show the same conclusion. These results are well in agreement with the analysis results of the crystallization kinetics. Moreover, the activation energies of the crystallization behavior and crosslinking behavior were obtained through the Kissinger method, and the values were about 152.7 kJ·mol^−1^ and 174.8 kJ·mol^−1^, respectively. Therefore, a conclusion can be drawn from the study that the activation energy of the crystallization behavior is less than that of the crosslinking behavior, indicating that the crystallization behavior more easily occurs than does the crosslinking reaction. As a result, the crystals of PEN-Ph can be self-crosslinked to form single-polymer composites.

## Figures and Tables

**Table 1:** Detailed data of crystallization kinetic parameters.

β	r	n	lnK_t_	lnK_c_	K_c_
5	0.969	2.12	−4.972	−0.994	0.370
10	0.967	2.22	−3.871	−0.387	0.679
15	0.979	2.28	−2.838	−0.189	0.828
20	0.979	2.23	−2.176	−0.109	0.897

**Table 2:** Detailed data of crosslinking activation energy at various conversions.

Conversions	Slopes	E_α_ (kJ·mol^−1^)
0.1	22.302	185.27
0.2	22.088	183.49
0.3	21.675	180.06
0.4	21.354	177.39
0.5	21.049	174.86
0.6	20.818	172.94
0.7	20.509	170.37
0.8	20.286	168.52
0.9	19.762	164.17
Average value		175.23

**Table 3:** Thermal and mechanical properties of amorphous PEN-Ph and PEN-Ph single-polymer composites.

Samples	Gel Fraction (%)	T_g_ (°C)	Tensile Strength (MPa)
Amorphous PEN-Ph	72.3	198.6	101.8
PEN-Ph single-polymer composites	73.3	204.6	111.0
